# When Size Matters: Surgical Challenges of Giant Rectal Foreign Bodies

**DOI:** 10.7759/cureus.115090

**Published:** 2026-08-24

**Authors:** Juan C Aguado-Valderrama, Santiago A Suárez-Gómez, Nathalia Melo-González, Fernando Escobar-Castañeda

**Affiliations:** 1 Department of Surgery, Pontificia Universidad Javeriana, Bogota, COL; 2 Department of Surgery, Maria Inmaculada State Hospital, Florencia, COL

**Keywords:** anal canal, colorectal surgery, colostomy, foreign bodies, general surgery, laparotomy, rectum, rural health services, wounds and injuries

## Abstract

Rectal foreign bodies are an uncommon but increasingly recognized presentation in emergency colorectal surgery. Object size - particularly transverse diameter - and rural, low-resource settings add complexity to both diagnosis and treatment. A 57-year-old man from a rural area of southern Colombia was referred to a regional hospital for a retained lithic rectal foreign body (a stone). Plain abdominal radiography showed a cone-shaped radiopaque object. After multiple failed transanal extraction attempts, exploratory laparotomy was performed. A conical stone approximately 15 cm in diameter at its base was found, impacted between the lower rectum and the sacral promontory. Extraction was carried out through an enterotomy at the rectosigmoid junction; during the maneuver, an upper rectal laceration occurred, which was repaired with a two-layer enterorrhaphy and protected with a loop sigmoid colostomy. The postoperative course was uneventful, and the patient was discharged in satisfactory condition.

With a basal diameter of approximately 15 cm, the object was at the upper end of the diameter range reported for rigid rectal foreign bodies requiring laparotomy. Because the pelvic outlet, rather than object length, represents the anatomical constraint to transanal extraction, the object's diameter and rigidity rendered transanal removal unfeasible. Timely escalation to laparotomy with protective diversion limited the extent of the rectal injury. Early recognition of transverse diameter, rigidity, and unfavorable morphology, together with a low threshold for surgical intervention, allows favorable outcomes even with limited infrastructure, where plain radiography and sound clinical judgment remain the mainstays of management.

## Introduction

Rectal foreign bodies are an uncommon but increasingly recognized presentation in emergency colorectal surgery. US statistics report an increase in the annual incidence from 1.2 per 100,000 in 2012 to 1.9 per 100,000 in 2021 [1]. Most cases result from intentional insertion, and delayed presentation - often driven by embarrassment or fear of judgment - is a critical contributor to complications, capable of turning a retained object into a surgical emergency involving perforation, obstruction, or sepsis [2-4]. Institutional series and cohort analyses document a rising number of emergency presentations over the past decade, predominantly among adult men, with a substantial proportion requiring operative management [2,5].

Contemporary management rests on a structured evaluation: a non-judgmental history, exclusion of peritonitis, early imaging to characterize the size and position of the object, and an extraction strategy that favors the transanal route when feasible and reserves operative intervention for its failure or for signs of perforation [3,4,6]. Most objects can be removed transanally, but a minority with unfavorable features - proximal migration, rigidity, or large size - require endoscopic or surgical assistance [7,8]. These challenges are amplified in rural settings, where geographic isolation, limited availability of computed tomography and endoscopy, and restricted access to subspecialty care shape decision-making. We report the case of an exceptionally large lithic rectal foreign body managed in a rural Colombian setting, in which the diameter and rigidity of the object dictated the need for laparotomy.

## Case presentation

**Figure 1:**
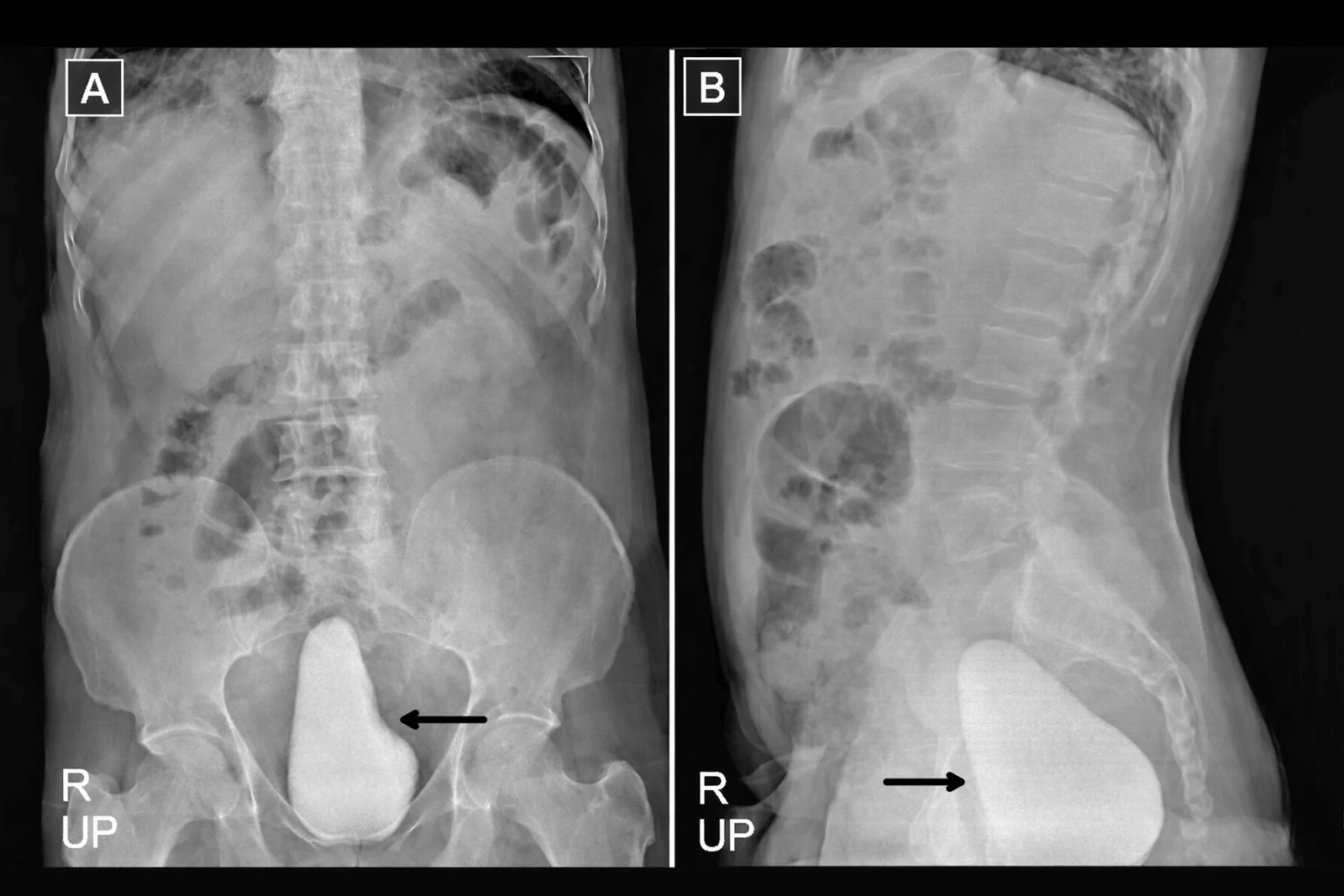
Plain abdominal radiographs showing a foreign body (arrows) impacted in the rectum. (A) Anteroposterior view. (B) Lateral view.

A 57-year-old man from a rural area of Southern Colombia presented with a one-day history of a retained rectal foreign body. He was initially assessed at a primary care center and referred to the regional hospital for definitive management. On admission, he was hemodynamically stable. Physical examination confirmed the presence of the rectal foreign body. Plain abdominal radiography showed a cone-shaped radiopaque object impacted in the rectum (Figure 1A, 1B), with approximate dimensions of 15 × 12 × 10 cm, which limited the feasibility of transanal extraction.

**Figure 2:**
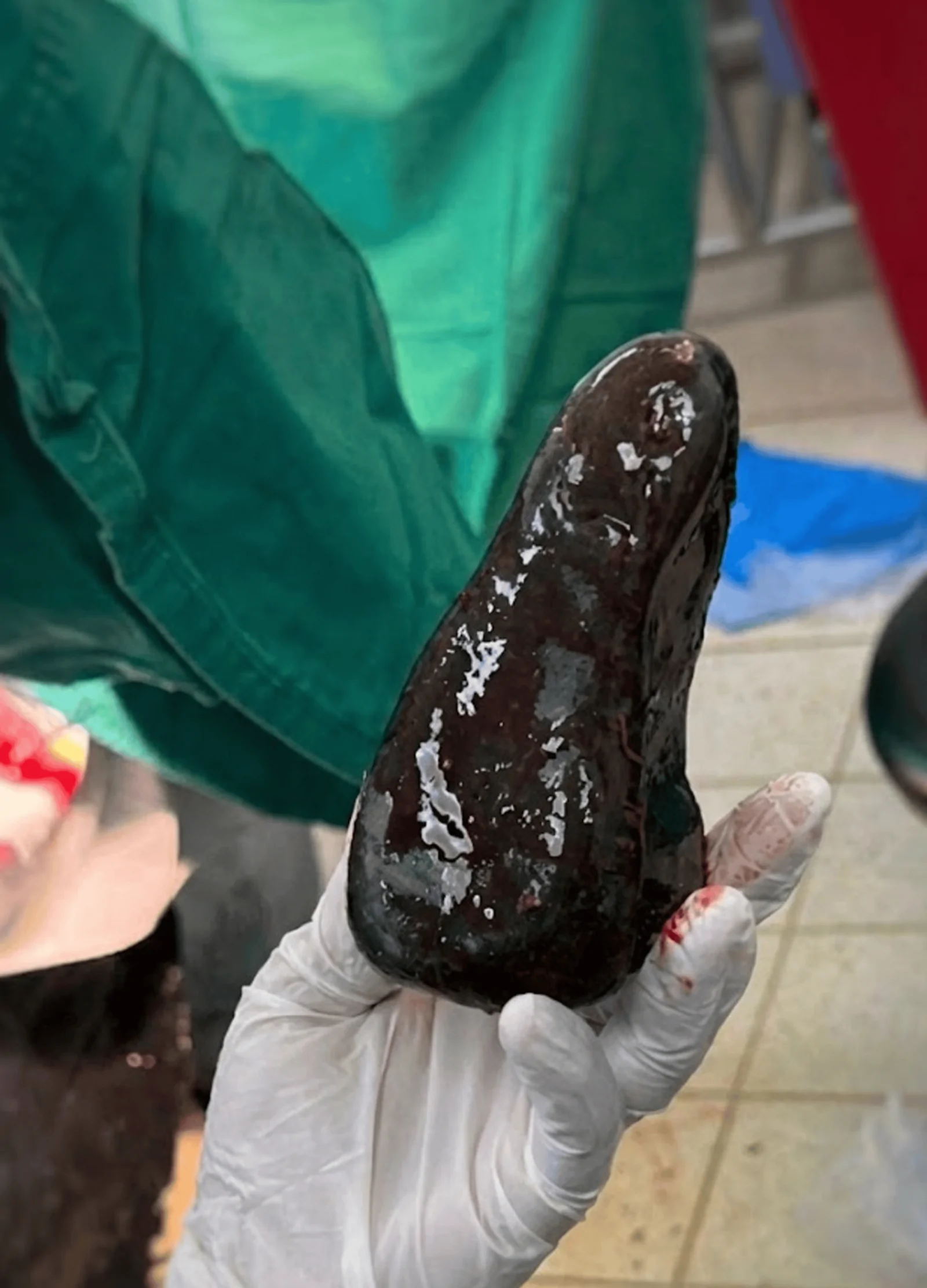
Intraoperative image of the rectal foreign body (stone) after surgical extraction. The final dimensions, measured by ruler, were 15 × 15 × 10 cm, with a diameter decreasing in size up to its upper portion of 6 cm.

Multiple attempts to mobilize and extract the object transanally were unsuccessful. Given its characteristics, urgent surgery was undertaken. Exploratory laparotomy was performed through an infraumbilical incision, initially under spinal anesthesia. Intraoperative assessment revealed a conical stone impacted between the lower rectum and the sacral promontory, with its wider base oriented toward the anal canal. The upper pole of the object was identified at the rectosigmoid junction, where an enterotomy was created to facilitate extraction. Conversion to general anesthesia with neuromuscular blockade allowed partial disimpaction and successful removal with a Rochester clamp. A cone-shaped stone measuring 15 cm in length, with a base approximately 15 cm in diameter and 6 cm at its upper portion, was extracted (Figure 2). The basal diameter measured after extraction exceeded the initial radiographic estimate, a discrepancy attributable to projection effects and to the difficulty of sizing an impacted object on plain films; the post-extraction caliper measurement of approximately 15 cm was therefore regarded as the definitive dimension. The final dimensions, measured by ruler, were 15x15x10 cm, with a diameter decreasing in size up to its upper portion of 6 cm.

During extraction, a laceration of the upper rectum occurred, attributable to the duration of impaction and to the size and rigidity of the object. The injury was repaired with a two-layer enterorrhaphy. Given its extent and the presence of macerated tissue edges, a protective loop sigmoid colostomy was constructed and matured with Brooke-type cardinal sutures.

Postoperatively, the patient received close in-hospital monitoring by the general surgery team, antibiotic prophylaxis with cefazolin and metronidazole, and standard colostomy care. His course was favorable, with no adverse events during hospitalization. He remained clinically stable and was discharged six days later in satisfactory condition, with outpatient follow-up.

## Discussion

Although most rectal foreign bodies are resolved with transanal extraction techniques, a subgroup requires operative intervention based on object characteristics. The present case exemplifies this subgroup: a large, rigid, lithic object that, after failed transanal attempts, required laparotomy with enterotomy and fecal diversion. Although neuromuscular relaxation diminishes reflex spasms of the anal sphincter and puborectal sling and thus increases the diameter of the anal canal and rectal compliance, prolonged surgical time (approximately 2 hours) and still an impossibility to remove the foreign object led the surgical team to perform a formal enterotomy. The foreign object also compressed the anatomical structures, and this prolonged mechanism caused a lacerative lesion. The clinical value of this case lies not in the rarity of rectal foreign bodies, a well-documented occurrence in the literature, but rather in the dimensions of the object and the complex surgical decisions its management necessitated

Current classification schemes stratify cases by location (low or high relative to the rectosigmoid junction), the presence of perforation or peritonitis, and object characteristics: size, shape, rigidity, and radiopacity [6,7,9]. In practice, one of the principal determinants of transanal feasibility is not the length of the object but its maximal transverse diameter relative to the pelvic outlet, which constitutes the limiting anatomical aperture; this has been described when the greatest diameter of an object exceeds the pelvic outlet [10]. In this case, a basal diameter of approximately 15 cm, together with lithic rigidity and impaction between the lower rectum and the sacral promontory, placed the object beyond the transanal range. Persisting with transanal maneuvers under such conditions increases the risk of iatrogenic injury to the rectal wall and of contamination, without improving the likelihood of success [3,9]. Less invasive alternatives to open surgery, including transanal minimally invasive surgery and laparoscopic-assisted retrieval, have been described for selected high-lying objects [11,12], but the object's diameter and rigidity rendered these approaches unsuitable and made open extraction necessary.

To situate the object within the published record, we compared its dimensions with those of large rectal foreign bodies reported in the literature (Table 1). Objects that reach greater lengths, such as rods or elongated prostheses, tend to remain narrow and are frequently removed transanally or endoscopically precisely because of their reduced diameter [4,13]. By contrast, the rigid, bulky objects that mandate laparotomy cluster at maximal transverse diameters of up to approximately 10 cm: an ovoid marble ornament with overall dimensions of 12 × 8 × 8 cm (transverse diameter 8 cm) that required pubic symphysiotomy [10], and an 11 × 10 cm rubber toy (transverse diameter 10 cm) managed with a Hartmann procedure, alongside a 7.5 cm baseball in a prior episode in the same patient [14]. With a basal diameter of nearly 15 cm, our patient's object lies at the upper end of this diameter spectrum for rigid objects, and its lithic composition precluded the deformation or controlled fragmentation that occasionally rescues a transanal attempt.

**Table 1:** Comparison of dimensions, composition, and extraction approach of large rectal foreign bodies reported in the literature. For rigid objects, the maximal transverse diameter—not the length—determines the feasibility of transanal extraction, since the pelvic outlet is the limiting aperture.

Object	Composition (rigidity)	Maximum dimensions	Extraction approach	Ref.
Stone (present case)	Mineral (lithic, rigid)	15 cm length × 15 cm basal diameter	Laparotomy + rectosigmoid enterotomy + loop colostomy	—
Ovoid marble ornament	Marble (lithic, rigid)	12 × 8 × 8 cm	Laparotomy + pubic symphysiotomy	[9]
Rubber toy	Rubber (semi-rigid)	11 × 10 cm	Laparotomy + Hartmann procedure	[13]
Baseball	Leather (rigid)	7.5 cm in diameter	Laparotomy	[13]
Silicone penile prosthesis	Silicone (flexible)	20 × 5 cm	Endoscopic	[12]
Steel rod	Steel (rigid)	77 cm in length	Not specified	[3]

The operative findings, a rectal laceration requiring two-layer enterorrhaphy and diversion, reflect mechanical injury from sustained intraluminal pressure, analogous to the pathophysiology of stercoral perforation [15] and distinct from the acute perforations produced by sharp or pointed objects [3,7,16]. The intraoperative finding of laceration validated the decision to perform a controlled enterotomy with protective colostomy rather than continued transanal manipulation, which would likely have aggravated tissue damage and contamination [7,9]. Conversion from spinal to general anesthesia with neuromuscular blockade facilitated safe extraction and repair, and underscores the value of anesthetic flexibility in complex cases.

In resource-limited settings, plain radiography was sufficient to characterize the morphology and dimensions of the object and to recognize the infeasibility of transanal extraction in the absence of computed tomography [8,17]. Postoperative management followed the principles of colorectal trauma: antibiotic prophylaxis with enteric coverage, close monitoring, and fecal diversion for a significant rectal injury with compromised edges [7,18,19]. Follow-up should address stoma function and planning for its reversal [3,7]. Although the literature emphasizes addressing underlying psychosocial factors, access to mental health services may be limited in rural Colombia, requiring individualized assessment and selective referral when indicated [3,17].

In summary, this case documents a lithic rectal foreign body whose transverse diameter lies at the upper end of what has been described for rigid objects requiring laparotomy (Table 1), and it illustrates a mechanism of injury from sustained pressure that was managed successfully in a resource-limited setting. Rather than proposing a new technique, it reinforces a principle: when the physical characteristics of an object exceed conventional algorithmic frameworks, early recognition of diameter and rigidity, together with fundamental surgical principles, remains decisive for the outcome.

## Conclusions

Rectal foreign bodies require individualized management according to object characteristics, location, clinical stability, and available resources. This case shows that when a rigid, large-diameter object exceeds the pelvic outlet, early recognition of this limitation and a low threshold for laparotomy with diversion prevent major rectal injury. In rural settings, plain radiography and sound clinical judgment are sufficient to guide safe decisions in the absence of advanced imaging. Future research should refine risk-stratification tools based on object diameter and rigidity and standardize criteria for stoma reversal.
